# Remittances, development and financialisation beyond the Global North

**DOI:** 10.1177/0308518X221088867

**Published:** 2022-03-27

**Authors:** Rahel Kunz, Julia Maisenbacher, Lekh Nath Paudel

**Affiliations:** IEP, 30656 Université de Lausanne Faculté des sciences sociales et politiques, Switzerland; IEP, Université de Lausanne Faculté des sciences sociales et politiques, Switzerland; IEP, Université de Lausanne Faculté des sciences sociales et politiques, Switzerland

**Keywords:** Remittances, Financialisation, Development, North-South relations, Subjectivities, Contestation

## Abstract

In the context of the global financial crisis and the crunch in development financing, remittances have become linked to the financial inclusion agenda in what has been termed as ‘financialization of remittances’ (FOR). This special issue brings together seven articles that analyze the socioculturally specific histories and the everyday manifestations of the FOR in the Caribbean, Central America, Colombia, Ghana, Mexico, Nepal and Senegal. The contributors engage in a transdisciplinary conversation, mobilizing insights from feminist, postcolonial, poststructural and political geography theories. They propose two majors shifts for financialisation analysis: towards an investigation beyond the global North and towards taking seriously failures, contradictions and contestations of financialisation processes. By doing so, the special issue contributes to financialization research in five major ways: to expose colonial legacies of remittances and their financialization; to challenge the supposedly neutral character of the FOR by revealing the caste, gendered and racialized power relations in financialization processes; to destabilizes the notion of the universal individual financial subject and show how multiple financial subjectivities are constituted in constellations; to document the complexities, ambiguities, contradictions and failures of financialization processes and the (everyday) contestations they face; and to show how remittances and their financialization are implicated in reconfiguring authorities, citizenship and social dynamics. The contributions propose relational understandings of financialization that conceptualize the co-constitution of economic, political and sociocultural dimensions of financialization across and beyond the North-South divide.

Since the late 1990s and against the backdrop of stagnating levels of official development aid, remittances – the money that migrant workers send to their families or communities of origin – have become heralded by the international development community as a key tool to promote development and reduce poverty. Officially recorded remittance flows^1^ to lower- and middle-income countries reached US$ 540 billion in 2020 and constitute more than 30% of GDP in countries such as Tonga or Lebanon (World Bank and KNOMAD, 2021). An estimated 800 million people worldwide are directly supported by remittances sent by over 200 million migrants.^2^ The mobilising of remittances for development has been referred to as the ‘global remittance trend’ (Kunz, 2011). In the wake of the global financial crisis, the international community has started to promote the linking of remittances to the global financial inclusion (FI) agenda. This agenda encourages access to financial education and services for low-income populations as a strategy for poverty reduction, development and women's empowerment (Gabor and Brooks, 2017; Rankin, 2013; Schwittay, 2011; Soederberg, 2014). It expands the focus from earlier emphasis on microfinance as a development tool to include the mainstreaming of financial institutions and the ‘broadening of the definition of “pro-poor” financial services to include savings and payment services alongside lending, targeting both households and small businesses’ (Gabor and Brooks, 2017: 426). The FI agenda redefines development through financial logics, reorients development interventions via financial markets and deepens financial infrastructure in the developing world, in what some term the financialisation of development (Carroll, 2015; Carroll and Jarvis, 2014; Gabor, 2018; Gabor and Brooks, 2017; Gabor and Brooks, 2017; Mader, 2014).

The linking of remittances to this FI agenda has been termed the financialisation of remittances (FOR). As part of the FOR, various international, governmental, non-governmental and private actors promote the formalisation, digitalisation and securitisation of remittances (Datta, 2017; Guermond and Samba Sylla, 2018; Hudson, 2008; Kunz, 2012; Zapata, 2013). The FOR represents a shift from earlier paradigms whereby remittances no longer necessarily contribute to development per se but need to be linked to financial logics, practices and (formal) institutions to do so effectively (Kunz et al., 2021). Remittances senders and receivers are targeted with financial education programs and financial services to encourage them to save and invest, to engage in risk-management and planning for the future, and to integrate them into the (formal) financial system (Ajefu and Ogebe, 2019). Novel instruments – such as diaspora bonds, microinsurance services, remittances transfer platforms and mobile banking tools – channel migrant money into finance and encourage pro-poor access to finance. Transnational households and diaspora communities have become framed as development actors and new clients for financial institutions. The overall objective of the FOR is to promote the ‘productive’ investment of remittances in financial products to foster disciplined financial subjects and development.

Yet, despite the fundamental importance of remittances for a big part of the global population and despite the increasing attention they receive from the international financial and development community, the linking of remittances to finance and FI has not received enough attention in the scholarly literature. This special issue aims to address this gap. The existing literature is dominated by economistic studies that analyse the phenomenon at the macro level, portraying it as a tool for development, economic growth and women's empowerment. These studies emphasise the beneficial impacts of remittances in terms of increasing household's access to and use of financial services for saving, self-sufficiency, consumption, health (Anzoategui et al., 2014; Demirgüç-Kunt et al., 2011; Labastida Tovar et al., 2014; Suri and Jack, 2016; Villasenor et al., 2016). As shown by Cross (2015), authors tend to take a problem-solving approach around the links between remittances, finance, economic growth and development. Challenging this narrow economistic and problem-solving approach, critical scholars emphasise the cultural political economy dimensions of the FOR and embed the phenomenon within broader logics of financialisation, understood as the expansion and the deepening of the influence of global finance in all areas of economic, political and social life (Cross, 2015; Datta, 2017; Guermond and Samba Sylla, 2018; Hudson, 2008; Hughes, 2011; Kunz, 2012; Mader, 2014; Zapata, 2013). They propose to understand the FOR as part of a broader global neoliberal project that aims to create new markets and to incorporate migrants and their families into commercial banking. And they highlight the problematic implications in terms of ‘the deepening entrenchment of the historical labour migration dynamic between sending communities and centres of capital’ (Cross 2015: 305).

What has so far received less attention, are the everyday manifestations of the links between development, remittances and finance; how these links manifest in gendered, racialised and socio-culturally specific ways; the ways in which these links impact on dynamics of debt and indebtedness; and the multiple forms of resistances and counter-movements to the FOR. Moreover, symptomatic of the broader Western-centric bias of the (critical) financialisation literature, few analyses focus on the links between migration, remittances and finance beyond the global North. This is surprising, given the central role remittances play in global and everyday finance. Moreover, remittances are a particularly fruitful heuristic to study financialisation trends that defy North–South binaries. Little has been written about the complex socio-culturally specific financial practices linked to remittances, such as informal financial transfer and investment activities, gift-giving practices, and rotating savings and credit associations. Studying context-specific financial practices linked to remittances seems crucial for a better and more holistic understanding of the complexity of global finance and for exploring alternative forms of agency and resistance to financialisation.

In order to address these gaps, this special issue proposes to shift financialisation analysis towards an investigation beyond the global North and towards taking seriously failure, contradiction and contestation. Seven contributors analyse how the FOR manifests in different contexts. They combine theoretical insights with detailed empirical analysis, focusing on various geographical contexts: the Caribbean, Central America, Colombia, Ghana, Mexico, Nepal and Senegal. The authors come from various disciplinary (geography, international relations and sociology) and geographical backgrounds. After summarising the contributions, this introduction presents the two shifts and the main contributions of the special issue to ongoing debates surrounding remittances, development and financialisation.

## Summaries of the contributions

Focusing on the everyday practices of negotiating the FOR, *Vincent Guermond* analyses forms of resistance to financialisation among remittance recipients in Ghana and Senegal. In ‘Contesting the financialisation of remittances: Repertoires of reluctance, refusal and dissent in Ghana and Senegal’, he sheds light on how remittance recipients respond to the FOR’s attempts to incorporate them into global finance. He finds that attempts to channel remittances away from mutual aid associations and deprive remittance recipients of their capacity to produce relational value face contestation. His contribution shows how concrete financial practices and subjectivities diverge from the ideal of the neoliberal self-disciplined subject.

In ‘“Cambiando el chip”: The gendered constellation of subjectivities of the financialisation of remittances in Mexico’ *Rahel Kunz and Brenda Ramirez* analyse the gendered manifestations of the FOR in Mexico. They propose the concept of ‘constellation of subjectivities’ to analyse the intersecting social dynamics of the governing arrangements of the FOR. They find that the FOR in Mexico creates and governs a gendered constellation of financial subjectivities with three dimensions: migrant men who are disciplined to use formal transfer channels and various financial services; remittance-receiving women who are encouraged to save and invest remittances; and non-transnational families who are under pressure to become included in financial institutions and who act as a constitutive outside in this constellation but are integral part of the FOR. Through this constellation, the FOR creates a gendered hierarchy between remittance-receiving and non-transnational families, pushing the latter towards informal financialisation. Thus, in Mexico, remittances are mobilised in gendered ways to increase the depth and scope of financialisation.

In ‘Racial Capitalism, Coloniality and the Financialization of Caribbean Remittances’, *Beverley Mullings* draws attention to the ways in which recent efforts in the Caribbean region to exercise control over financial flows threaten the continuity and accessibility of remittances. She examines three initiatives – the rationale and unsuccessful efforts to create a government diaspora bond issue in Haiti and Jamaica, the failed proposal to tax Caribbean remittances, and the enforcement of Anti-Money Laundering and Countering the Financing of Terrorism policies throughout the region. Her analysis reveals the coloniality of emerging structures of financial control and how these create new economies of dispossession reliant on finance that enact new forms of racial subjugation and dispossession. She details the new battle lines that emerge as financial institutions, financial elites and imperializing states become attuned to the value and implicit autonomy that remittances generate in the Caribbean.

*Lekh Nath Paudel* explores the historical antecedents of the FOR in the context of rural Nepal in ‘Remittances and the reconfiguration of rural finance in Nepal (1900–1960)’. He proposes a temporal and spatial shift to investigate the role of remittances in the reconfiguration of rural finance and social hierarchies. Drawing on the concept of ‘debt-reign’ as a political formation allows him to analyse the links between statecraft technologies, debt and caste relations, and remittances. His analysis shows the disruptive and transformative power of remittances shaping the (re)negotiation of, and emancipation from, debt relations, and the reconfiguration of social hierarchies. He also details the ways in which colonial monetisation and reconfiguration of rural finance prepared the ground for the introduction of contemporary financial infrastructures such as credit cooperatives and banks. His analysis exposes colonial legacies and historicises the FOR.

*Araby Smyth* draws on ethnographic research in an indigenous village in Oaxaca, Mexico, to analyse the everyday experiences of women in remittance receiving communities in ‘Challenging the financialization of remittances agenda through Indigenous women's practices in Oaxaca’. She shifts the focus on remittances towards detailed empirical analysis of how people navigate the FOR in their lives. Inspired by feminist and postcolonial scholarship, her contribution challenges two individualist and dichotomous assumptions underlying the FI agenda: that remittances spending is either productive or not, and that women are empowered or not through linking remittances to financial institutions. She shows how everyday practices of remittance-receiving women transcend the empowered/disempowered and the formal/informal dichotomy on which the FOR is based.

‘The financialization of remittances and the individualization of development: A new power geometry of global development’ by *Hannes Warnecke-Berger* aims to transcend a narrow economistic and problem-solving perspective on remittances and FI to show the global power dimensions involved in the FOR. He explores the reconfiguration of social settings, actors and strategies in relation to remittances to reveal the structural effects of the FOR. His contribution highlights how the FOR produces a shift in the political economy of development in three ways. First, the FOR amplifies the micro-macro divergence inherent in remittances. Given that remittances are mediated through currency exchange rates, individual migrants benefit from larger exchange rate differentials and from the subordinated role of their home economies within the world economy. Deepening the financial ‘development’ impact of remittances then goes hand in hand with cementing global inequality. Second, certain economic and political elites in remittance-receiving societies are able to organise access to remittances through financialisation and emancipate from national political control, which fosters elite rule. Finally, the FOR contributes to consolidate an individualised notion of development.

*Gisela Zapata* explores the relationship between the FOR and diaspora policies in Colombia in ‘Diaspora engagement policies and transnational financialisation in Colombia’. She shows how the Colombian state has expanded political and social rights and launched symbolic initiatives to link remittance senders to their home country over the past 20 years. These diaspora policies aim to capture and maintain the financial connections of diaspora members to their homeland. In Colombia, the FOR is embedded in a broader strategy of redefining transnational state membership which includes both embracing diaspora members through the extension of social and political rights and tapping into their connections to global circuits of capital and finance. This model of diaspora engagement feeds into financialised models of development.

## Beyond the global North

In recent years, financialisation has gained much attention. A growing body of literature analyses the causes and consequences of the growing importance of finance for the economic system and everyday life. It scrutinises how the expansion of financial markets affects economic activities, everyday practices and the formation of financial subjects (Mader et al., 2020; van der Zwan, 2014). For a long time, the financialisation literature focused mostly on Anglo-Saxon contexts. More recently, financialisation processes beyond the Global North started to receive attention. A nascent body of literature analyses how financialisation considered as originating in Western industrialised economies affects the South (Bortz and Kaltenbrunner, 2017; James, 2017; Karacimen, 2014). This literature focuses on the consequences of the rise of finance-led capitalism in the Global North on developing countries, such as increasing individual indebtedness, rise of private equity and increased austerity measures (Becker et al., 2010). Thereby, financialisation is defined as a structural global shift which affects political economies unevenly and creates new dependencies. The focus is on conceptualising financialisation in developing and emerging economies – for example, as subordinated (Powell, 2013) – and the institutions, politics and local factors shaping financialisation. Moreover, studies document how developing countries cope with financialisation (Bonizzi, 2013; Karwowski and Stockhammer, 2017) and how financialisation influences everyday life and consumer behaviour in developing countries (Gonzalez, 2015; Lai, 2017).

This special issue contributes to this nascent literature aimed at decentring financialisation research from the Anglo-American economies and the global North. We explicitly frame our focus as ‘beyond the global North’ to make a number of contributions. The various case studies yield insights into the multiplicity and socio-culturally specific histories and manifestations of financialisation processes beyond the usual focus on the North. Yet, as Pollard has warned, the aim is not to head South in order to universalise or overgeneralise from the experiences of the global South (Pollard, 2013). The contributors mobilise insights from feminist, postcolonial, poststructural and political geography approaches in order to ‘inform the production of more complex, less partial geographies of financialization’ (Pollard, 2013). Rather than reproducing binary analytical separations, we draw attention to what goes on beyond the North and to what is connected to the North but has been silenced by the current bias in financialisation research. In addition to expanding the focus of financialisation research, we also aim to transcend the binary thinking that treats financialisation in the North and South as analytically separate phenomena. We suggest that North and South must be considered as two sides of the same coin. The analysis of remittances and their financialisation is a fruitful terrain to move towards a more relational understanding of how North and South are interrelated and co-constitutive. This approach also allows us to challenge underlying assumptions of existing accounts of the FOR and of financialisation more broadly.

For example, the contributions by *Mullings*, *Paudel*, *Warnecke-Berger* and *Zapata* show how remittances have become an important economic, political and financial asset for governments, financial institutions and elite actors across the global South, increasing their influence over these flows. They highlight that the FOR is embedded in North–South relations and shapes dynamics of global inequality and local and global geometries of power. *Mullings*, *Paudel* and *Smyth* highlight the colonial legacies of remittances, financial institutions and relations of extraction in the Caribbean, Mexico and Nepal. *Mullings* investigates in detail the ongoing coloniality between the Caribbean and the US and the efforts of political interests in the US to link remittances to national security concerns in order to control these flows. Various contributors challenge underlying assumptions of existing financialisation narratives. Through a focus on micro level practices linked to the FOR in Oaxaca, *Smyth* for example challenges two dichotomies underlying the financialisation literature and the global development agenda regarding the productive spending of remittances and women's empowerment linked to remittances and financial institutions. *Kunz and Ramirez*, *Mullings* and *Paudel* challenge the supposedly neutral character of the FOR by revealing the caste, gendered, racialised power dimensions involved in financialisation processes in the Caribbean, Mexico and Nepal. *Guermond, Kunz and Ramirez* and *Smyth* challenge the ideal of the individual empowered neoliberal financial subject which lies at the heart of the FOR and financialisation more broadly. To provide a more adequate account of how the FOR manifests through interdependent gendered subjectivities, *Kunz and Ramirez* propose the concept of constellation of subjectivities. They also challenge the idea that the FOR involves mostly transnational families by demonstrating how non-transnational families are impacted by, and participate in, informal and formal financialisation processes.

## Failure, contradictions and contestation

This special issue contributes to the critical research that documents the contradictions and failures of financialisation and the dissent and resistance it faces. This research challenges conceptualisation of financialisation as linear, continuous, universal and coherent scripts and processes. Instead, authors investigate how financialisation fails and pay attention to the everyday realities and bottom-up struggles in response to financialisation. For example, Brett Christophers suggests that financialisation both as a concept and a phenomenon faces analytic, theoretic, strategic, optic and empiric limits (Christophers, 2015). Nick Bernards investigates how financialisation dynamics and the formation of financial markets are contradictory and sometimes fail (Bernards, 2019). Analysing the political struggles in the financialisation of housing, Desiree Fields demonstrates how financialisation can be contingent and ‘incomplete’ and encourages readers to understand financialisation not as a monolithic and inevitable process, but as one characterized by resistance from without and contradiction from within (Fields, 2017). In her analysis of care service companies in the UK, Amy Horton shows how care workers refuse to perform as financial subjects and thus ‘constrain the expansion of financial discipline’ and ‘constrain financialisation’ (Horton, 2022). In addition, Maisenbacher (forthcoming) draws on a historical materialist approach to analyse structural political economy limits to the FOR (Maisenbacher, forthcoming), cited here. She identifies how a non-financialised cooperative FI model of migrant workers has emerged in Kenya with some positive effects for labour and development.

The contributions of this special issue show the ambiguity, complexity and failure of financialisation processes linked to remittances through detailed case studies. Their analysis shows how finance impacts the everyday lives of families in complex, contradictory and gendered and racialised ways. *Mullings*, *Warnecke-Berger* and *Zapata* show that the FOR does not necessarily fulfil its development promise. Instead, it increases the influence on remittances of government, financial institutions and elite actors and contributes to individualise and financialise development. In Colombia, the FOR is part of a broader process of the redefinition of (transnational) state membership in which contradictory empowering and disciplining elements co-exist (*Zapata*). In Mexico, the FOR does not necessarily promote inclusion into the formal financial system but imbricates formal and informal finance in gendered ways (*Kunz and Ramirez*). Focusing on the analysis of everyday practices of managing remittances, *Guermond* and *Smyth* reveal how financial services and financial reasoning shape people's everyday experiences, practices, norms and socio-economic arrangements. In the context of Oaxaca, *Smyth* highlights how local knowledge systems, economic networks and money management techniques mediate the experience of the FOR of remittance recipients. *Guermond* analyses how the FOR in Ghana and Senegal leads to variegated subjectivities of contestation that differ from the neoliberal ideal of the self-disciplined and risk-averse financial subject. Practices of contestation, disruption and refusal manifest in the creation of alternative and collective financial spaces which follow different financial logics compared to those promoted by the FOR. *Paudel* documents how remittances in rural Nepal play a complex and contradictory role disrupting and reconfiguring social and financial hierarchies and increasing indebtedness for some while allowing others to reduce their debt, creating new power inequalities.

Overall, this special issue offers insights into how the FOR and financialisation processes more broadly manifest in multiple ways and involve contradictions, failures and resistance. Bringing together various case studies allows us to unravel the multi-layered entanglements of the FOR and to historicise and re-embed this phenomenon in cultural, political and economic contexts beyond the global North. Taking remittances as a starting point highlights the imbrications and mutual constitution of economic, political and sociocultural dimensions of financialisation dynamics and allows us to develop conceptual tools for the analysis of financialisation across and beyond the North–South divide. The contributions speak to several current debates, such as the literature on the migration-remittances-development nexus; FI, financial subjectivities and development; global finance and de-/financialisation tendencies; transnational households; and indebtedness. They also dialogue with everyday IPE research that analyses how broader political economy developments interact with everyday social dynamics, decisions, desires and contestations regarding financialisation. Yet, much work remains to fully grasp the multiple ways in which financialisation, remittances and development interact.

## Notes

1. Official numbers do not include ‘informal’ remittances, thought to be substantial. Yet, the increase in official remittances figures is also influenced by the increased capacity to register such flows.

2. https://refugeesmigrants.un.org/sites/default/files/stocktaking_ifad.pdf (accessed 18 March 2022).
